# Risk Factors of Intravenous Immunoglobulin Resistance in Children With Kawasaki Disease: A Meta-Analysis of Case-Control Studies

**DOI:** 10.3389/fped.2020.00187

**Published:** 2020-04-21

**Authors:** Gengying Liu, Shunyu Wang, Zhongdong Du

**Affiliations:** Department of Cardiology, Beijing Children's Hospital, Capital Medical University, National Center for Children's Health, Beijing, China

**Keywords:** Kawasaki disease, intravenous immunoglobulin resistance, risk factors, meta-analysis, children

## Abstract

Previous studies have shown that children with Kawasaki disease (KD) who fail to respond to intravenous immunoglobulin (IVIG) therapy are at higher risk of developing coronary artery lesions (CALs). We aimed to conduct a meta-analysis to uncover the risk factors associated with IVIG resistance in children with KD. PubMed, Embase, and Cochrane Library databases were searched up to 31st October 2019, and 23 case-control studies were finally eligible, enrolling 2,053 patients of IVIG resistance and 16,635 patients of IVIG sensitivity. Potential factors were comprehensively analyzed by using stata15 software with a standard meta-analysis procedure and consequently found that in addition to patients with polymorphous rash or swelling of extremities symptoms had a tendency to be non-responders, IVIG resistance was more likely to occur in patients with severe anemia, hypoalbuminemia, decreased baseline platelet count, and elevated levels of erythrocyte sedimentation rate (ESR), total bilirubin, alanine aminotransferase (ALT) and neutrophils percentage. Particularly, male sex, hyponatraemia, increased aspartate aminotransferase (AST), and C-reactive protein (CRP) were confirmed as the risk factors favor IVIG resistance in Mongoloids from Asia countries, but not in Caucasians from non-Asia regions. In summary, we report several risk factors relevant to IVIG resistance in children with KD, which may provide guidance for the prediction of IVIG resistance. But a proposing of an optimal prediction system with high specificity and sensitivity needs further studies because of confounding factors.

## Introduction

Kawasaki disease (KD) is an acute medium-sized vasculitis of unknown etiology, characterized by persistent fever and five typical clinical manifestations, including swelling of extremities, polymorphous rash, cervical lymphadenopathy, oral lesions, and bilateral conjunctivitis (1). It predominantly affects children younger than 5 years old and results in coronary artery lesions (CALs) such as ectasias or aneurysms in 25% of untreated patients (2), considering one of the most common cause of acquired heart disease in children in many countries (3). Timely treatment with intravenous immunoglobulin (IVIG) and oral aspirin has reduced the prevalence of CALs from 25% to about 4% (4). However, 10%–20% of patients with KD fail to respond or develop recrudescent fever 36–48 h after the first dose of IVIG (5), which are termed as IVIG resistance. Previous studies have shown that resistant patients are at higher risk of developing CALs (5, 6), and addition use of glucocorticoid or cyclosporine have been confirmed effectively reduce the incidence of CALs in children predicted with IVIG resistance before treatment according to different randomized clinical trails (7, 8). Therefore, it is of importance to predict targeted patients who will be IVIG non-responders so that they can benefit from the more aggressive therapy regarding CALs prevention.

The pathological development of KD is a systemic inflammatory process, during which the severity degree of inflammation is reflected in the duration of fever, more severe or clearer diagnostic clinical manifestations, and higherly activated laboratory parameters such as hemoglobin, baseline platelet count, percentage of neutrophils, erythrocyte sedimentation rate (ESR), C-reactive protein (CRP), total bilirubin, albumin, alanine aminotransferase (ALT), aspartate aminotransferase (AST), serum sodium and other inflammatory biomarkers, which once have been reported to differ remarkably between IVIG non-responders and responders before IVIG infusion. But these factors have no clear consensus on predicting IVIG resistance so far and the conflicting data may derived from ethnic and genetic backgrounds (9, 10).

This study was designed to perform a meta-analysis to identified risk factors associated with IVIG resistance in patients with KD. It may be helpful for prediction of IVIG non-responsiveness.

## Materials and Methods

This meta-analysis was conducted in accordance with the guidelines of the Preferred Reporting Items for Systematic Reviews and Meta-analyses Statement (11).

### Database Search

We searched databases including PubMed, Embase and Cochrane Library up to 31st October 2019 by using Medical Subject Headings (MeSH) terms or Emtree thesaurus terms combined with keywords, the search strategy was [“Mucocutaneous lymph node syndrome” OR “Kawasaki disease”] AND [“IVIG resistance” OR “IVIG non-responsiveness” OR “IVIG unresponsiveness”]. The language was restricted to English and a manual search was conducted using reference lists of original articles for further articles of interest.

### Inclusion and Exclusion Criteria

#### Criteria for Inclusion

①Case-control study or cohort study; ②All subjects were children (aged 0–18 years) diagnosed with KD according to Japanese diagnostic criteria (12) or the 2017 American Heart Association common standards (4) and received IVIG infusion in the cumulative dose of 2 g/kg plus oral aspirin for the initial therapy; ③Odds ratio (OR) and 95% confidence interval (CI) provided for categorical variables in the original data or mean and standard deviation provided for continuous variables, and all the data provided were measured at the time of admission; ④Clear description of statistical methods and correct statistical analyses.

#### Criteria for Exclusion

①Animal studies; ②Reviews, duplicates, case reports, meta-analyses, conference abstracts or unpublished literatures; OR and 95% CI were not provided for categorical variables or mean and standard deviation were not provided directly or indirectly for continuous variables.

### Data Extraction and Quality Assessment

The data were independently gathered by 2 investigators on the basis of a predefined standard form. The data extracted from the studies included such details as the first author, publication year, region, sample size, clinical symptoms and laboratory indicators of cases and controls. We assessed quality of every included study with respect to cases and controls selection, comparability, and exposure using the Newcastle-Ottawa Quality Assessment Scale (NOS) for case-control or cohort studies (Supplementary Material). which has a total score of 9 stars, a study that awards ≥7 stars is considered high methodological quality, ≤ 3 as low quality, and 3–7 as moderate.

### Statistical Analysis

Data analysis was performed by using Stata15 software. Cochrane Q test and *I*^2^ statistic were calculated to assess heterogeneity across studies. If the studies were shown to be homogeneous with *P* ≥ 0.10 and *I*^2^ <50%, the fixed effects model was selected, otherwise, the random effects model was applied (13). The pooled effects were presented with OR and corresponding 2-tailed 95% CI for dichotomous variables, or weighted mean difference (WMD) and corresponding 2-tailed 95% CI for continuous variables. The significance of the pooled effects were determined by the Z-test, all *P*-values were 2-tailed and a *P* < 0.05 was considered statistical significantly. Sensitivity analysis was conducted by omitting a single study involved in the meta-analysis in turn to identify the potential influence of each individual data on the pooled effects and to confirm that our results were not driven by any single study. Publication bias was estimated via funnel plot and Egger's test (14), a *P* > 0.05 was considered no significantly publication bias.

## Results

### Search Results

**Figure 1:**
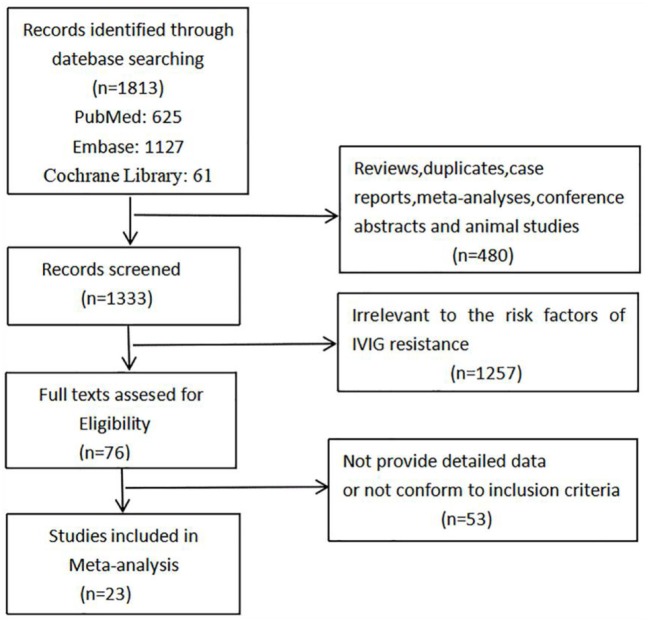
Flow chart of studies selection process.

Of 1,813 articles initially searched, 480 reviews, duplicates, case reports, meta-analyses, conference abstracts and animal studies were firstly removed. After screening the titles and abstracts, 1,257 studies that irrelevant to the risk factors of IVIG resistance were excluded, and another 53 studies that did not provide detailed origin data or not in accordance with our inclusion criteria were excluded after assessing the full text. Finally, 23 studies were found to conform to our specific inclusion criteria and were included consequently in our meta-analysis. The studies selection process is shown in Figure 1.

### Study Characteristics and Quality Assessment

All the 23 included studies (15–37) were case-control studies, the general characteristics were summarized in Table 1. A total of 18,688 patients were enrolled, of which 2,053 were assigned in the IVIG-resistant group and 16,635 in the IVIG-sensitive group. These studies were conducted in different ethnic populations and different regions, including Mongoloids from Asia (Japan, China, Chinese Taiwan, South Korea, Thailand) and Caucasians from non-Asia (Spain, Israel, USA). The quality assessment found that all the included studies awarded ≥7 stars (Table 2), indicating that the studies are of high methodological quality and persuasive.

**Table 1:** Characteristics of included studies in the meta-analysis.

**References**	**Region**	**Gender (male/female)**	**Number of patients (*****n*****)**		**Resistant rate (%)**
			**IVIG-resistant**	**IVIG-sensitive**	
Arane et al. (15)	Israel	239/43	52	230	18.44
Chantasiriwan et al. (16)	Thailand	141/76	26	191	11.98
Egami et al. (17)	Japan	184/136	41	279	14.7
Fu et al. (18)	China	746/431	211	966	17.93
Gamez-Gonzalez et al. (19)	Japan	244/175	101	318	24.11
Kim et al. (20)	South Korea	401/302	118	585	16.79
Kim et al. (21)	South Korea	–/–	524	4627	10.17
Kim et al. (22)	South Korea	84/51	22	113	16.3
Kuo et al. (23)	Chinese Taiwan	93/38	20	111	15.27
Loomba et al. (24)	USA	105/77	58	124	31.87
Nakagama et al. (25)	Japan	109/62	54	117	31.58
Park et al. (26)	South Korea	161/148	30	279	9.71
Sanchez-Manubens et al. (27)	Spain	238/161	67	332	16.79
Sano et al. (28)	Japan	59/53	22	90	19.64
Sato et al. (29)	Japan	62/43	21	84	20
Sleeper et al. (30)	USA	125/73	27	171	13.64
Tan et al. (31)	China	3,269/2,009	348	4,929	6.59
Tang et al. (32)	China	584/326	46	864	5.05
Yang et al. (33)	China	827/533	78	1282	5.74
Lin et al. (34)	Chinese Taiwan	107/74	22	159	13.84
Lee et al. (35)	South Korea	47/44	11	80	12.09
Bar-Meir et al. (36)	Israel	202/110	42	270	13.46
Kobayashi et al. (37)	Japan	231/315	112	434	20.51

**Table 2:** Quality assessment of included studies by NOS.

**References**	**Selection**	**Comparability**	**Exposure**	**NOS scores**
Arane et al. (15)	⋆⋆⋆	⋆⋆	⋆⋆	7
Bar-Meir et al. (36)	⋆⋆⋆⋆	⋆⋆	⋆⋆⋆	9
Chantasiriwan et al. (16)	⋆⋆⋆⋆	⋆⋆	⋆⋆	8
Egami et al. (17)	⋆⋆⋆	⋆⋆	⋆⋆	7
Fu et al. (18)	⋆⋆⋆	⋆⋆	⋆⋆	7
Gamez-Gonzalez et al. (19)	⋆⋆⋆	⋆⋆	⋆⋆	8
Kim et al. (20)	⋆⋆⋆	⋆⋆	⋆⋆	7
Kim et al. (21)	⋆⋆⋆⋆	⋆⋆	⋆⋆	8
Kim et al. (22)	⋆⋆⋆	⋆⋆	⋆⋆	7
Kobayashi et al. (37)	⋆⋆⋆⋆	⋆⋆	⋆⋆	8
Kuo et al. (23)	⋆⋆⋆⋆	⋆⋆	⋆⋆⋆	9
Lee et al. (35)	⋆⋆⋆	⋆⋆	⋆⋆	7
Lin et al. (34)	⋆⋆⋆	⋆⋆	⋆⋆⋆	8
Loomba et al. (24)	⋆⋆⋆	⋆⋆	⋆⋆	7
Nakagama et al. (25)	⋆⋆⋆	⋆⋆	⋆⋆	7
Park et al. (26)	⋆⋆⋆⋆	⋆⋆	⋆⋆	8
Sanchez-Manubens et al. (27)	⋆⋆⋆	⋆⋆	⋆⋆⋆	7
Sano et al. (28)	⋆⋆⋆	⋆⋆	⋆⋆⋆	8
Sato et al. (29)	⋆⋆⋆	⋆⋆	⋆⋆	7
Sleeper et al. (30)	⋆⋆⋆⋆	⋆⋆	⋆⋆⋆	9
Tan et al. (31)	⋆⋆⋆	⋆⋆	⋆⋆	8
Tang et al. (32)	⋆⋆⋆	⋆⋆	⋆⋆	7
Yang et al. (33)	⋆⋆⋆⋆	⋆⋆	⋆⋆	8

### Risk Factors

**Figure 2:**
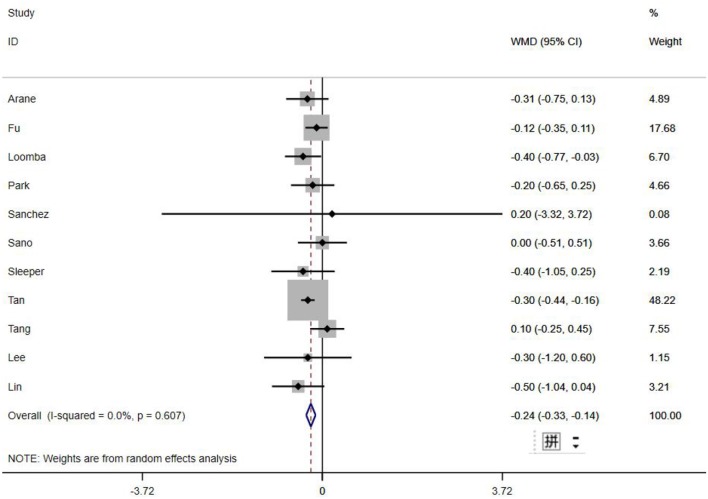
Pooled weighted mean difference for IVIG resistance by hemoglobin (g/dL).

**Figure 3:**
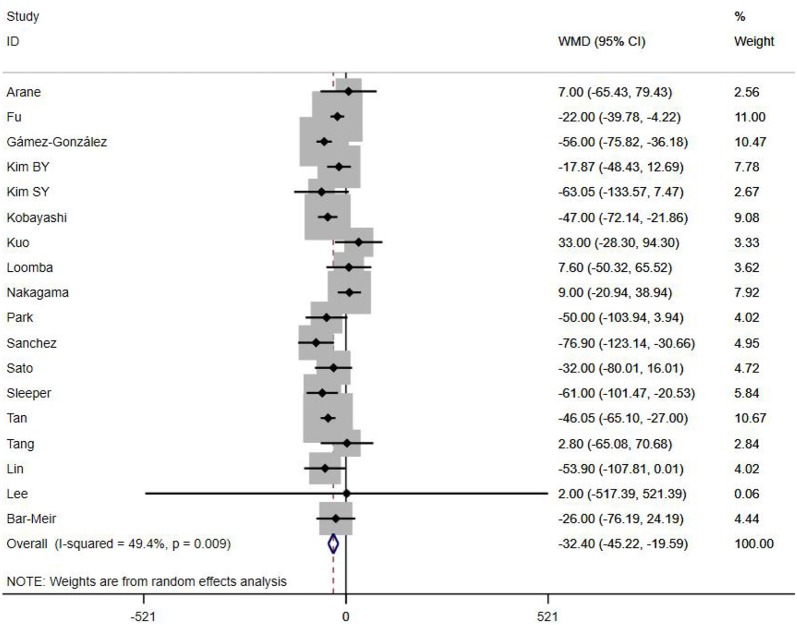
Pooled weighted mean difference for IVIG resistance by baseline platelet count (×10^9^/L).

**Figure 4:**
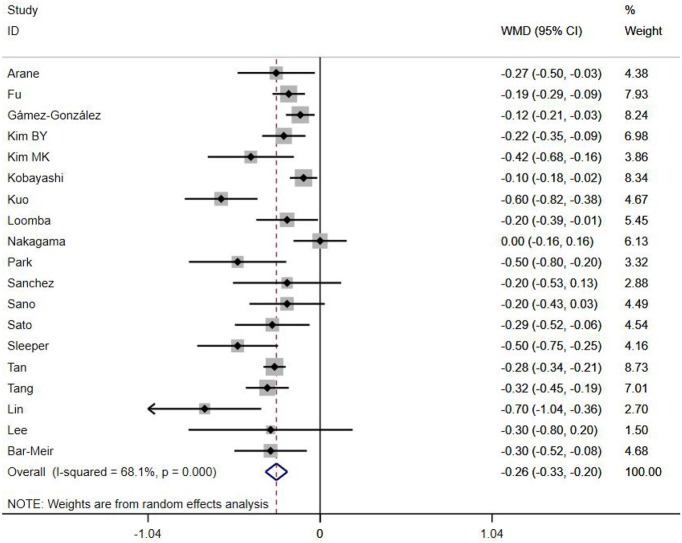
Pooled weighted mean difference for IVIG resistance by albumin (g/L).

**Figure 5:**
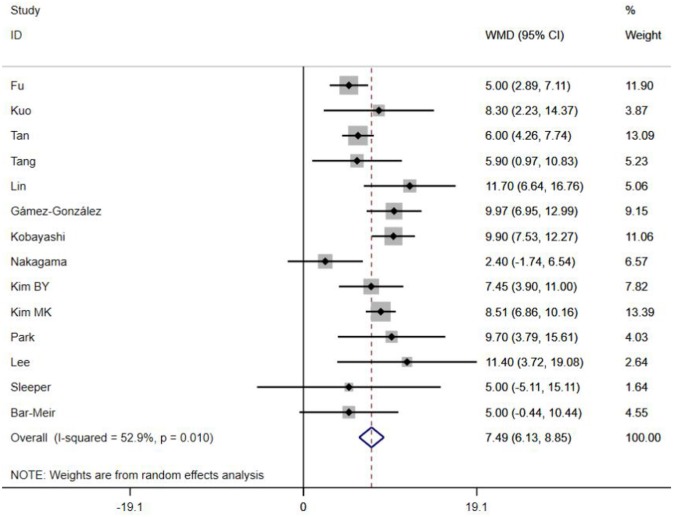
Pooled weighted mean difference for IVIG resistance by percentage of neutrophils (%).

**Figure 6:**
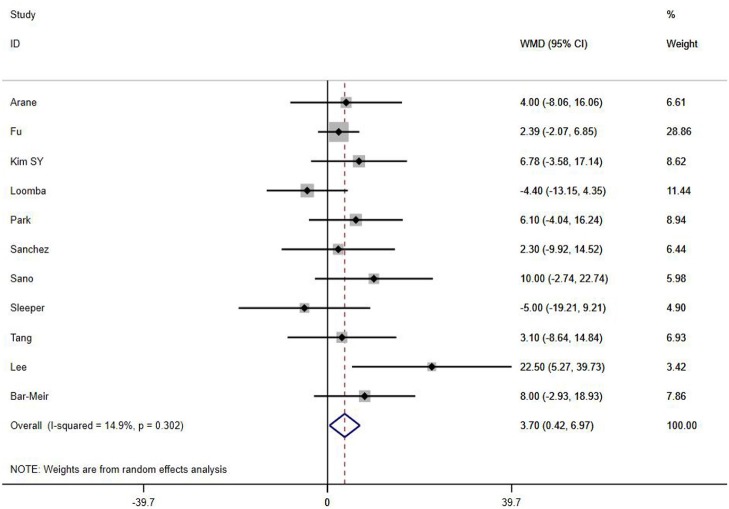
Pooled weighted mean difference for IVIG resistance by ESR (mm/h).

A total of 13 clinical and laboratory indicators were found associated with IVIG resistance, the results were summarized in Table 3. Our meta-analysis revealed that male patients were more likely to be IVIG non-responders (OR = 1.19, 95% CI: 1.01 to 1.42, *P* = 0.043) than females, and there was an increase in the association of both swelling of extremities (OR = 1.25, 95% CI: 1.01 to 1.54, *P* = 0.040) and polymorphous rash (OR = 1.56, 95% CI: 1.20 to 2.02, *P* < 0.001) with the odds of IVIG resistance. In terms of laboratory parameters, IVIG non-responders had significantly lower hemoglobin (Figure 2, WMD = −0.24, 95% CI: −0.33 to −0.14, *P* < 0.001), baseline platelet count (Figure 3, WMD = −32.4, 95% CI: −45.22 to −19.59, *P* < 0.001), albumin (Figure 4, WMD = −0.26, 95% CI: 0.33 to −0.20, *P* < 0.001) and serum sodium (WMD = −1.24, 95% CI = −1.63 to −0.85, *P* < 0.001) values than responders, while percentage of neutrophils (Figure 5, WMD = 7.49, 95% CI: 6.13 to 8.85, *P* < 0.001), ESR (Figure 6, WMD = 3.70, 95% CI: 0.42 to 6.97, *P* = 0.018), CRP (WMD = 2.21, 95% CI = 1.54–2.89, *P* < 0.001), total bilirubin (WMD = 0.49, 95% CI: 0.42 to 0.57, *P* < 0.001), AST(WMD = 42.27, 95% CI: 25.54 to 59.00, *P* < 0.001), and ALT (WMD = 39.76, 95% CI: 26.65 to 52.87, *P* < 0.001) values were significantly higher than responders.

**Table 3:** Meta-analysis of risk factors for IVIG resistant KD patients.

**Factors**	**Studies number**	**Heterogeneity**		**Pooled effect**		**Egger's test (*P*)**
		***I*^**2**^ (%)**	***P***	**OR/WMD (95% CI)**	***P***	
Male	20	36.60	0.052	1.19 (1.01, 1.42)	0.043	0.922
Swelling of extremities	7	36.40	0.138	1.25 (1.01, 1.54)	0.040	0.901
polymorphous rash	7	2.10	0.409	1.56 (1.20, 2.02)	<0.001	0.646
Hemoglobin (g/dL)	11	0.00	0.607	−0.24 (−0.33, −0.14)	<0.001	0.748
PLT count (×10^9^/L)	18	49.40	0.009	−32.40 (−45.22, −19.59)	<0.001	0.431
Neutrophils (%)	14	52.90	0.010	7.49 (6.13, 8.85)	<0.001	0.804
ESR (mm/h)	11	14.90	0.302	3.70 (0.42, 6.97)	0.018	0.260
CRP (mg/L)	20	64.70	0.000	2.21 (1.54, 2.89)	<0.001	0.148
Total bilirubin (mg/dL)	12	30.10	0.152	0.49 (0.42, 0.57)	<0.001	0.095
Albumin (g/L)	19	68.10	0.000	−0.26 (−0.33, −0.20)	<0.001	0.094
ALT	18	66.30	0.000	39.76 (26.65, 52.87)	<0.001	0.714
AST	19	84.10	0.000	42.27 (25.54, 59.00)	<0.001	0.630
Sodium (mmol/L)	16	68.70	0.000	−1.24 (−1.63, −0.85)	<0.001	0.549

We also identified several factors that were not relevant to IVIG resistance, including WBC (WMD = 0.33, 95% CI: −0.04 to 0.70, *P* = 0.078), age (WMD = 0.39, 95% CI: −0.90 to 1.68, *P* = 0.554), conjunctivitis (OR = 0.85, 95% CI: 0.62 to 1.15, *P* = 0.277), oral lesions (OR = 0.76, 95% CI: 0.56 to 1.03, *P* = 0.081) and cervical lymphadenopathy (OR = 0.99, 95% CI: 0.70 to 1.42, *P* = 0.974).

### Subgroup Analyses

Subgroups were selected based on different ethnic populations from different regions (Asian or non-Asian). All the high-risk factors with significant heterogeneity and more than 10 studies enrolled were analyzed, including male, baseline platelet count, percentage of neutrophils, CRP, albumin, ALT, AST, and serum sodium. It turned out that there were significant ethnicity-specific and region-specific differences in factors of male sex, elevation of CRP and AST, and decreased serum sodium, but not in baseline platelet count, percentage of neutrophils, albumin and ALT.

The summary WMDs for baseline platelet count were −21.87 (95% CI: −41.32, −2.42) and −35.73 (95% CI −68.47, −3.00) in Asian and non-Asian populations (P difference = 0.151). The WMDs for neutrophils percentage were 7.67 (95% CI: 6.22, 9.12) and 5.00 (95% CI: 0.21, 9.79) for the studies in Asian and non-Asian populations (P difference = 0.085). The WMDs for albumin and ALT were −0.25 (95% CI: −0.33, −0.18) vs. −0.29 (95% CI: −0.39, −0.18), and 45.98 (95% CI: 29.41, 62.56) vs. 21.30 (95% CI: 5.83, 36.77), respectively, for the studies in Asian and non-Asian populations, with P difference = 0.430 and 0.183.

**Figure 7:**
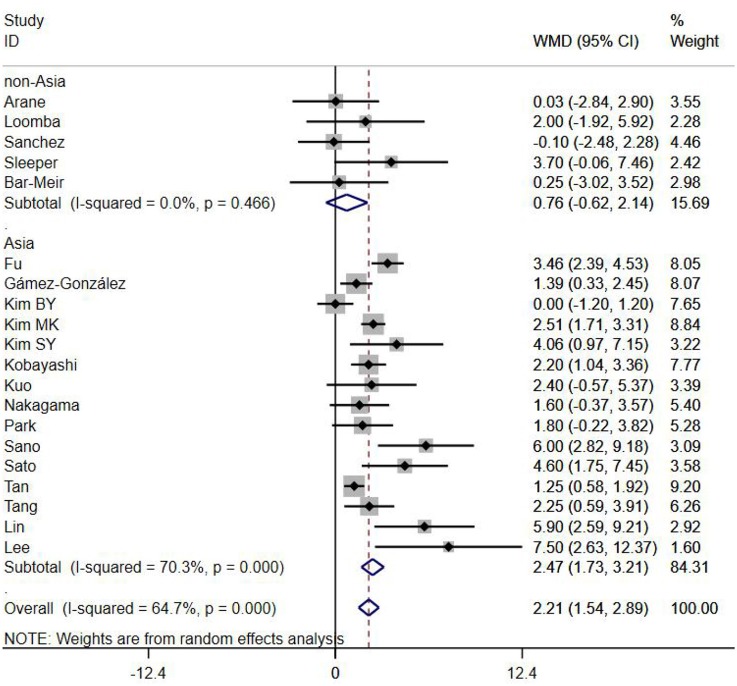
Subgroup analysis for IVIG resistance by CRP (mg/L).

**Figure 8:**
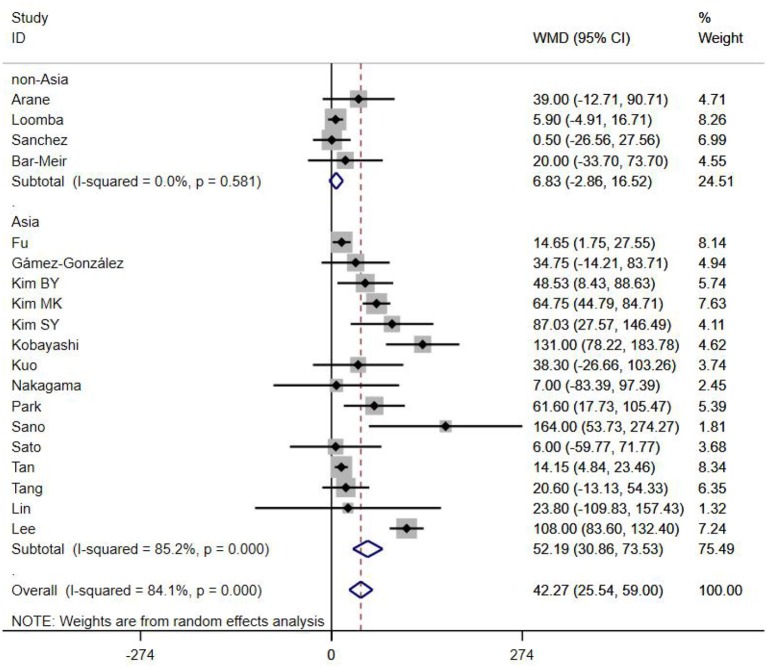
Subgroup analysis for IVIG resistance by AST (IU/L).

**Figure 9:**
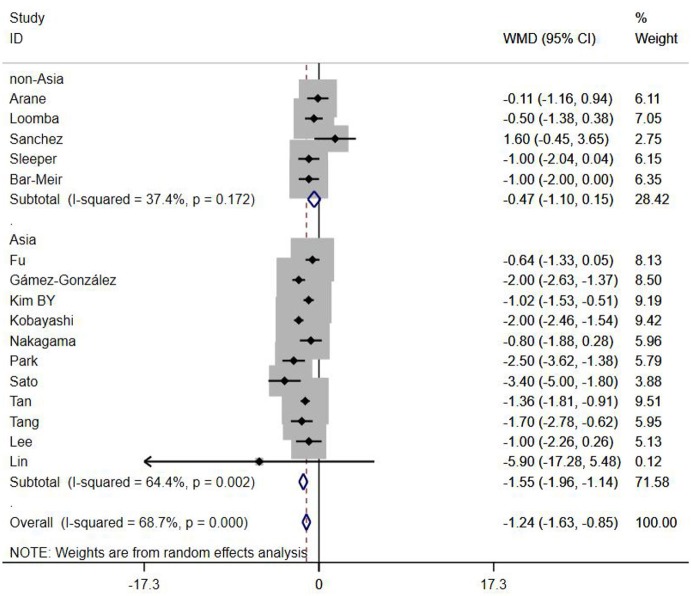
Subgroup analysis for IVIG resistance by sodium (mmol/L).

It is noteworthy that Asian male patients were more likely to be non-responders (*P* = 0.027), but there was no significant difference between responders and non-responders in non-Asian patients (*P* = 0.507). As well as male sex, Asian patients with higher CRP, AST and lower serum sodium were more likely to be IVIG non-responders (All *P* < 0.001%), and no significant differences were observed between responders and non-responders in Caucasians from non-Asia regions (All *P* > 0.05) (Figures 7–9).

### Sensitivity Analysis

**Figure 10:**
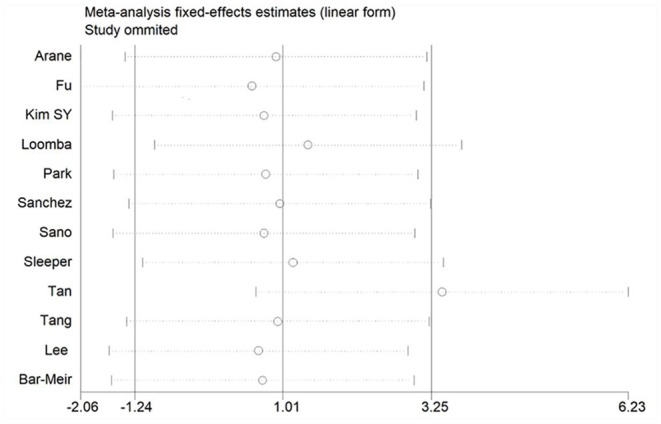
Sensitivity analysis of ESR.

If there is significant heterogeneity among studies, addition or reduction of any one study may lead to remarkable change in results, so we used sensitivity analysis to verify the reliability of the meta-analysis findings. In addition to the discovery that Tan's study (31) had a great impact on pooled effect in the meta-analysis of ESR as shown in Figure 10 (The ESR data of this study was omitted in this meta-analysis), there was no significant change in the pooled OR and WMD after every single study was omitted, this indicated that our results were convinced because of good stability and not driving by any single study.

### Estimation of Publication Bias

**Figure 11:**
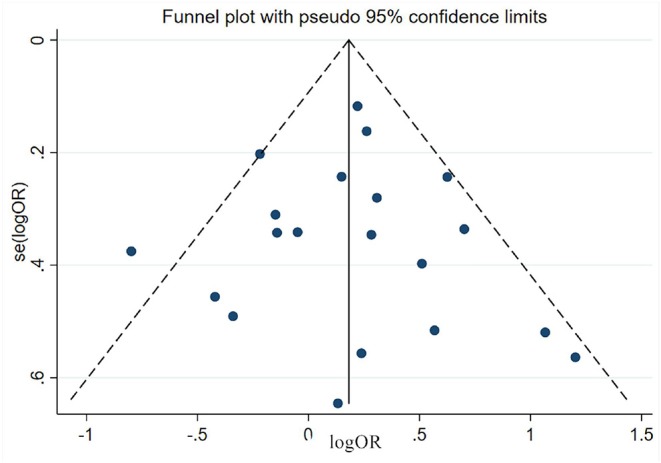
The funnel plot of male factor.

Publication bias was estimated via funnel plot and Egger's test. All the funnel plots showed generally symmetrical (e.g., the funnel plot of male sex shown in Figure 11) and all the *p*-value of Egger's test >0.05 (Table 3), which mean no significant publication bias was found in the meta-analyses of each risk factor.

## Discussion

KD is an infection-related immune-mediated systemic inflammation, although the clinical phenotype of KD varies across individuals, the pathogenesis is basically definite. After an infection of unknown agents, immune cells (especially T cells) are activated. Then the hyperactivated immune cells produce massive cytokines, leading to a cytokine imbalance associated with further endothelial cell injury (38), CALs may begin to develop and progress in the early phase of the inflammation, so early prediction of IVIG resistance through clinical manifestations and laboratory parameters is indeed a wise method for the severely affected patients who need early intensive treatment.

In the present meta-analysis, we included 23 studies from Japan, South Korea, China, Chinese Taiwan, Thailand, Spain, Israel and the United States of America, to analyze the relationships between non-responsiveness and clinical or laboratory indicators that are associated with inflammation. Also, we have discussed the indicators between different ethnicities based on various genetic backgrounds. The results showed that in addition to patients with polymorphous rash or swelling of extremities symptoms had a tendency to be non-responders, IVIG resistance was more likely to occur in patients with severe anemia, hypoalbuminemia, decreased baseline platelet count, and increased ESR, total bilirubin, ALT and neutrophils percentage. In particular, male sex, hyponatraemia, elevated levels of AST and CRP were confirmed as the risk factors favor IVIG resistance in Mongoloid patients from Asia countries, but not in Caucasians from non-Asia regions. No significant difference was found in WBC count, age, conjunctivitis, oral lesions and cervical lymphadenopathy between responders and non-responders. Subgroup analyses showed that some factors differed by ethnicity. The difference of gender, CRP, AST, and serum sodium between Asian and non-Asian patients were significant. Male Asian patients with increased CRP, AST and decreased serum sodium were more likely to be IVIG non-responders, while the same phenomenons were not observed in Caucasians from non-Asia regions.

Changes in these indicators can be explained by the pathophysiology of KD. KD inflammatory reaction changes the redox state of serum, with elevated inducible nitric oxide synthase (39), which would alter the homeostasis of red blood cells (RBCs) and result in a type of premature in these cells that lead to anemia and thrombus. RBC aging, inflammation and thrombus inevitably result in increased ESR. Intensive inflammation and immune reaction lead to serious vascular permeability and liver damage, resulting in albumin leakage and transaminases elevation. It is confirmed that cytokines such as plasma IL-6 and tumor necrosis factor-α (TNF-α) participate in inflammation of KD in the acute phase and were markedly increased in IVIG non-responders compared with responders (40–42), and the release of ADH is promoted by IL-6 and TNF-αduring inflammation (43), so the probable pathophysiological for hyponatremia may be relevant to inappropriate release of ADH. Overall, anemia, decreased serum albumin and sodium, elevated levels of neutrophils percentage and acute phase reactants such as ESR and CRP largely represent a more severe degree of inflammation and intensive immune response.

There are several limitations of this study should be noted. Firstly, the presence of confounding factors may reduce the accuracy of prediction and treatment guidance. The intensity of KD inflammation gradually increases in the acute stage and reach the peak, then decreases and enters to the convalescent stage (44), and the immune reaction before the peak may be responsible for tissue cell injury while immune reaction after the peak may be responsible for tissue cell repair (38), so inflammatory indices change throughout this process over time. Obviously, fever duration is a confounding factor predicting IVIG resistance. Besides, some laboratory values varied according to age, personal immunity and organ or tissues involvement in individuals. Secondly, the definition of IVIG resistance is not completely uniform in all the included studies, the observation periods after IVIG are different (24, 36, or 36–48 h). Thirdly, there are some differences in the infusion modalities of IVIG, some patients treated with IVIG 2 g/kg as a single infusion, while others received IVIG 1 g/kg for 2 days or 400 mg/kg for 5 consecutive days, this may lead to slightly different sensitivity to IVIG due to dose-response effects. As well as limitations, our meta-analysis also has significant aspects. We included various studies involving different ethnic populations from all over the world to ensure the applicability of our findings and to investigate a wide range of risk factors for IVIG resistance, and we had a sufficient sample size to carry out Egger's test for most factors and found no apparent publication bias.

In conclusion, some parameters were demonstrated associated with IVIG resistance, and clinicians should aware an increased likelihood that the patient may fail to respond to initial IVIG therapy when such factors present, but further studies are needed because of confounding factors in data analysis.

## Data Availability Statement

All datasets analyzed for this study are included in the article/Supplementary Material.

## Author Contributions

GL and ZD designed the study. GL contributed to literatures search, data collection, statistical analysis, and drafting the manuscript. SW contributed to literature search and data collection. ZD performed manuscript review. All authors have read and approved the content of the final manuscript.

## Conflict of Interest

The authors declare that the research was conducted in the absence of any commercial or financial relationships that could be construed as a potential conflict of interest.
